# Enhancing the Thermoelectric Performance of n-Type PbTe via Mn Doping

**DOI:** 10.3390/ma18051029

**Published:** 2025-02-26

**Authors:** Tingting Chen, Yaqi Shao, Ruilin Feng, Junxiang Zhang, Qidong Wang, Yanan Dong, Hongan Ma, Bing Sun, Dongwei Ao

**Affiliations:** 1 School of Physics and Electronic Information, Weifang University, Weifang 261061, China; 2 National Key Lab of Superhard Materials, Jilin University, Changchun 130012, China; 3 School of Machinery and Automation, Weifang University, Weifang 261061, China

**Keywords:** n-type PbTe, Mn doping, effective mass, strengthened point defect scattering

## Abstract

Significant strides have been made in enhancing the thermoelectric properties of p-type PbTe alloys, whereas the thermoelectric performance of n-type alloys lags behind that of p-type alloys, primarily owing to the difficulty of improving their Seebeck coefficient via band convergence. In this work, Mn was introduced into the n-type Pb_0.985_Sb_0.015_Te alloy, and Mn doping increases the absolute value of the Seebeck coefficient significantly by increasing the effective mass and reducing carrier concentration, resulting in a higher power factor of 20.8 μW/K^2^cm being achieved for 0.5% Mn-doped sample at 573 K. Additionally, the decrease in electronic thermal conductivity, combined with the reduction in lattice thermal conductivity caused by the strengthened point defect scattering, leads to a significant decrease in the total thermal conductivity of the sample. And the lowest total lattice thermal conductivity of 1.16 Wm^−1^K^−1^ for a 2.0% Mn-doped sample has been achieved at 773 K. In the end, a maximum *zT* of 1.0 (773 K) and *zT_ave_* of 0.62 (323–773 K) are attained in 1.0% Mn-doped Pb_0.985_Sb_0.015_Te alloy.

## 1. Introduction

Thermoelectric generators (TEGs) are solid-state semiconductor devices that convert heat directly into electrical energy [1,2]. The maximum power generation efficiency of TEG, *η*, is determined by $\eta_{max}=\frac{T_{h}-T_{c}}{T_{h}}\frac{\sqrt{1+zT_{ave}}-1}{\sqrt{1+zT_{ave}}+T_{c}/T_{h}}\;$, where *T_h_* and *T_c_* are the hot-side and cold-side temperatures, and *zT_ave_* is the average figure of merit calculated by $zT_{ave}=\frac{1}{T_{h}-T_{c}}\int_{T_{c}}^{T_{h}}zTdT$. *zT* is the dimensionless figure of merit of thermoelectric materials and defined by *zT* = *S^2^T*/*ρ*(*κ_e_ + κ_L_*), where *T* is the absolute temperature, *S* is the Seebeck coefficient, *ρ* is the electrical resistivity, *κ_e_* is the electronic thermal conductivity, and *κ_L_* is the lattice thermal conductivity, respectively [3,4,5,6,7]. Therefore, to achieve high power generation efficiency in TEG, it is necessary for thermoelectric materials to exhibit high *zT* values over a broad temperature range. Attaining a high *zT* necessitates a combination of a high Seebeck coefficient, low electrical resistivity, and low thermal conductivity. However, due to the strong interdependence among these parameters, it is difficult to optimize thermoelectric performance by optimizing just a single parameter.

PbTe exhibits excellent thermoelectric performance in the medium temperature region due to its complex electronic band structure, high crystal structure symmetry, and strong phonon anharmonicity [8,9]. Matched n- and p-type materials with high thermoelectric performance are the basis for high conversion efficiency and good service performance of thermoelectric devices. With the in-depth study, the performance of p-type PbTe materials has been significantly enhanced, whereas the performance of n-type PbTe materials remains relatively lower in comparison [10,11,12,13,14]. In the last several years, various approaches have been adopted to enhance the *zT* of n-type PbTe alloys. For example, Luo et al. synthesized Pb_0.975_Ga_0.025_Te-0.25%ZnTe with a high *zT_ave_* of approximately 1.26 (400–873 K) [15]. This high performance is attributed to the increased *n* by inducing Ga_2_Te_3_ precipitation and reduced *κ_L_* caused by softer phonon modes. And the PbTe_0.987_S_0.01_I_0.003_ alloy exhibited a *zT* value of 1.7 at 750 K, attributed to the mitigation of Pb vacancies and a substantial enhancement in carrier mobility [14]. In addition, *zT* values of 1.8 were achieved in PbTe_1-y_I_y_-3%Sb and PbTe-4%InSb alloys, attributing to the dual-site point defects and multiphase nanostructure engineering, respectively [16,17]. In the above-mentioned reports, the values of *zT* and *zT_ave_* of n-type PbTe alloy materials have been improved greatly; however, these values are still inferior to those of p-type alloys. This is mainly because it is difficult to optimize the *S* in n-type PbTe through band degeneracy. The absolute value of the *S* is proportional to the density of states effective mass ($m_{d}^{*}$), given by $m_{d}^{*}=N_{v}^{2/3}m_{b}^{*}$, with $m_{b}^{*}$ representing the local band effective mass [18]. The energy gap between the L and Σ bands in the valence band of PbTe is approximately 0.18 eV, facilitating the convergence of multiple bands. However, the energy gap between the corresponding bands is 0.45 eV, making it difficult to narrow the distance between them, and band degeneracy cannot be used to enhance the *S* of n-type PbTe [8]. Therefore, it is necessary to explore new strategies in band engineering for improving the *zT* of n-type PbTe alloys.

Recently, band flattening has emerged as a significant strategy for optimizing the electrical properties of thermoelectric materials, as it can distinctly enlarge the effective mass, thereby enhancing the *S*, which has been applied to various thermoelectric alloys, including SnTe, PbTe, InSe, GeTe, SnSe, and BiCuSeO [19,20,21,22,23,24,25,26,27]. Typically, the *zT_max_* values of SnTe and BiCuSeO increase substantially from ~0.6 to ~1.1 at 823 K and ~0.5 to ~1.2 at 723 K through flattening conduction bands, respectively [22,25]. Moreover, it has been proven that doping S, Mn, or Gd can significantly induce conduction band flattening in n-type PbTe [21,26,27]. However, previous studies frequently incorporated the introduction of nano-second phases, which may enhance thermoelectric performance and also add complexity to the system. Hence, in this work, on the basis of our previous research where Sb doping was used to regulate major carrier type and carrier concentration, the element manganese (Mn) is introduced without the emergence of nano-second phases in the samples. This enabled a clearer exhibition of the direct impact of Mn doping and also further optimization of the thermoelectric properties of n-type PbTe. Mn doping promotes the flattening of energy bands and enhances the effective mass, resulting in an elevation in the absolute value of the Seebeck coefficient. Despite the increase in resistivity due to the decrease in carrier concentration and mobility, the power factor is enhanced. In addition, the doped Mn also acts as an effective point defect phonon scattering center, thereby leading to the reduction in lattice thermal conductivity. Consequently, a peak *zT* of 1.00 at 773 K and a *zT_ave_* value of 0.62 over the temperature range of 323–773 K were obtained in a 1.0% Mn-doped n-type PbTe alloy.

The following are the Materials and Methods, Results, and Conclusions sections. The Materials and Methods section outlines the materials utilized in the experiments, the sample preparation methods employed, the characterization techniques adopted, and the methods used for testing and calculating electrical and thermal parameters. The Results section comprehensively presents the experimental outcomes, including an in-depth analysis of phase structure, microstructure, element distribution, electrical properties, and thermal properties. The impact of Mn doping on thermoelectric characteristics is specifically discussed. The Conclusions section summarizes the main findings and provides useful references for future research directions.

## 2. Materials and Methods

High-purity elements Pb (Trillion Metals Co., Ltd., Beijing, China, 99.99%), Te (Alfa, 99.9999%), Sb (Trillion Metals Co., Ltd., 99.99%), and Mn (Alfa, MA, USA, 99.9%) were precisely weighed in the ratio Pb_0.985−x_Sb_0.015_Mn_x_Te, with x varying as 0.5%, 1.0%, and 2.0%, and loaded into evacuated and flame-sealed quartz tubes. The tubes were gradually heated to 1073 K, held for 2 h, and then raised to 1273 K for 6 h, aiming at synthesizing the main phase. They were slowly cooled to 873 K over a period of 2 h, held at that temperature for 12 h, and finally cooled naturally to achieve a more uniform chemical composition within the sample. The ingots were pulverized and hot-pressed at 823 K for 60 min under 40 MPa, yielding highly dandified alloys with densities exceeding 97% of the theoretical value.

The crystal phases were recorded by X-ray diffraction (XRD, Rigaku, Tokyo, Japan, Smartlab3KW). The microstructure and chemical composition were characterized using field-emission scanning electron microscopy (SEM, JSM-6701F, JEOL, Tokyo, Japan,) coupled with EDS. The weight losses of samples were analyzed using a thermogravimetric analyzer (TGA, SDT650, TA Instruments, New Castle, DE, USA). The room-temperature Hall coefficient (*R_H_*) of each sample was measured using the Van der Pauw method through a homemade system under a magnetic field of ±0.6 T and an electrical current of 100 mA. The carrier concentration (*n*) was calculated using the formula *n* = 1/*eR_H_*, where *e* represents the electronic charge. The resitivity and Seebeck coefficient were measured using the LSR-3 instrument. The thermal conductivity was obtained through *κ_tot_* = *λC_p_d_E_*, where the thermal diffusivity (*λ*) was measured using a laser thermal conductivity meter (DLF1200), the heat capacity (*C_p_*) was calculated using the formula *C_p_* = 3.07 + 4.7 × (T-300) × 10^−4^ [28], and the experimental density (*d_E_*) was obtained using the Archimedes’ method with the Deiartts ES-E120D instrument and calculated through the formula $d_{E}=m_{1}d_{0}/\left(m_{1}-m_{2}\right)$. In this formula, *m*_1_ represents the mass of the sample in air, *m_2_* represents the mass of the sample when submerged in water, and *d_0_* represents the density of water (which is 0.996511 g/cm³ at 27 °C). Based on these parameters, the *zT* was determined. The uncertainties of the measurements and the calculated parameters are 6% for *ρ*, 8% for *S*, 11% for *κ_tot_*, and 19% for *zT*, respectively [29].

## 3. Results

**Figure 1:**
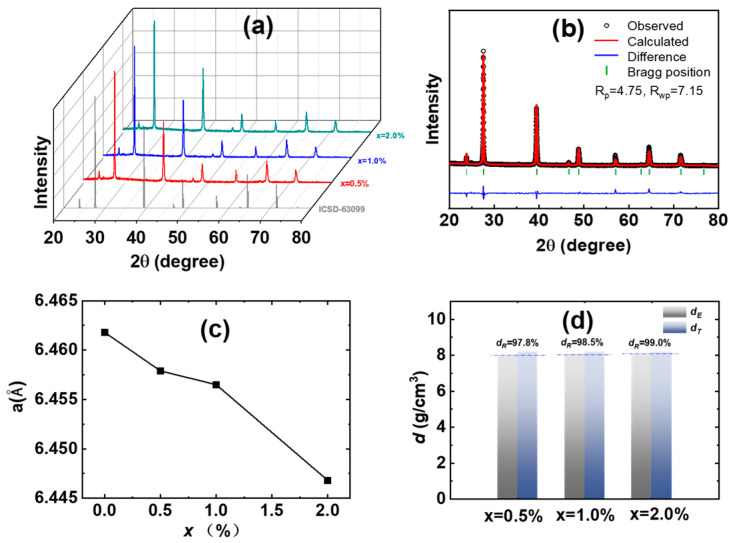
(**a**) XRD patterns of Pb_0.985−x_Sb_0.015_Mn_x_Te bulk alloys. (**b**) XRD patterns with Rietveld refinement of x = 1.0% sample. (**c**) Lattice parameters and (**d**) experimental densities (*d_E_*) and theoretical densities (*d_T_*) and relative densities (*d_R_*) of Pb_0.985−x_Sb_0.015_Mn_x_Te bulk alloys.

The XRD results of Pb_0.985−x_Sb_0.015_Mn_x_Te alloys are presented in Figure 1a. It is evident that almost all diffraction peaks are well matched with the PbTe structure (space group: Fm-3m, ICSD: 63099), and no significant impurity peaks are observed. The powder XRD data were employed to determine the theoretical density (*d_T_*) and lattice parameters through the Rietveld refinement method. The theoretical density is calculated using the formula $d_{T}=ZM/\left(N_{A}a^{2}\right)$, where *Z* represents the number of atoms in the unit cell, *M* represents the molar mass of the atom, *N_A_* represents Avogadro’s constant, and *a* represents the lattice parameters. And the relative density is calculated by $d_{R}=d_{E}/d_{T}\times 100\%$. These results are illustrated in Figure 1c for lattice parameters and Figure 1d for densities, respectively. The lattice parameters gradually decrease from 6.458 Å to 6.447 Å in Pb_0.985−x_Sb_0.015_Mn_x_Te (x = 0.5–2.0%), which is due to the ionic radius of Mn^2+^ (≈0.76 Å, Coordination: VI) being smaller than that of Pb^2+^ (≈1.19 Å, Coordination: VI) [30,31,32].

**Figure 2:**
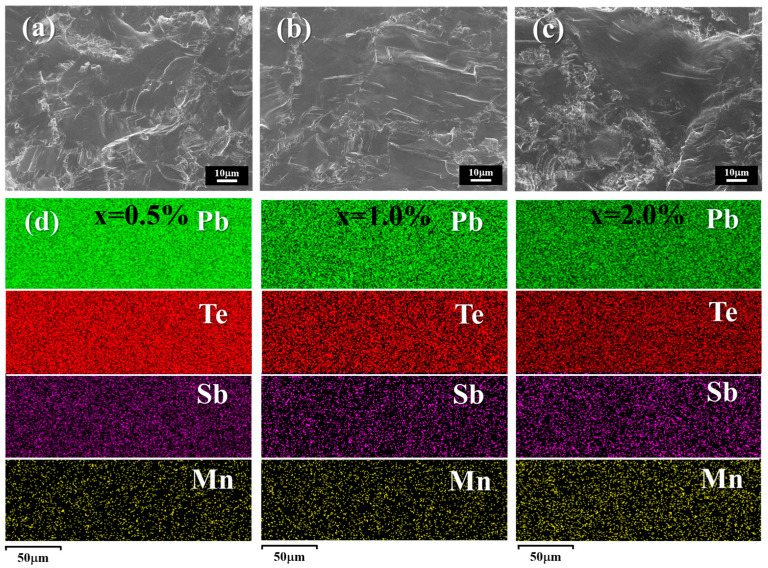
The SEM images of the cross-sectional surface for Pb_0.985−x_Sb_0.015_Mn_x_Te alloys for (**a**) x = 0.5%, (**b**) x = 1.0%, and (**c**) x = 2.0%. (**d**) The EDS mapping of the polished surface for Pb_0.985−x_Sb_0.015_Mn_x_Te alloys.

The cross-sectional surface microstructures of the Pb_0.985−x_Sb_0.015_Mn_x_Te alloys are displayed in Figure 2a–c. A notable observation is the absence of pores on the surface of all samples, aligning well with the high density presented in Figure 1d. To study the elemental composition and distribution of the as-synthesized sample, EDS element mapping of the polished surface was conducted, and the results are presented in Figure 2d. Evidently, the presence of Pb, Te, Sb, and Mn is discernible solely, with an absence of any element-concentrated zones, which aligns with the findings obtained from XRD analysis.

**Figure 3:**
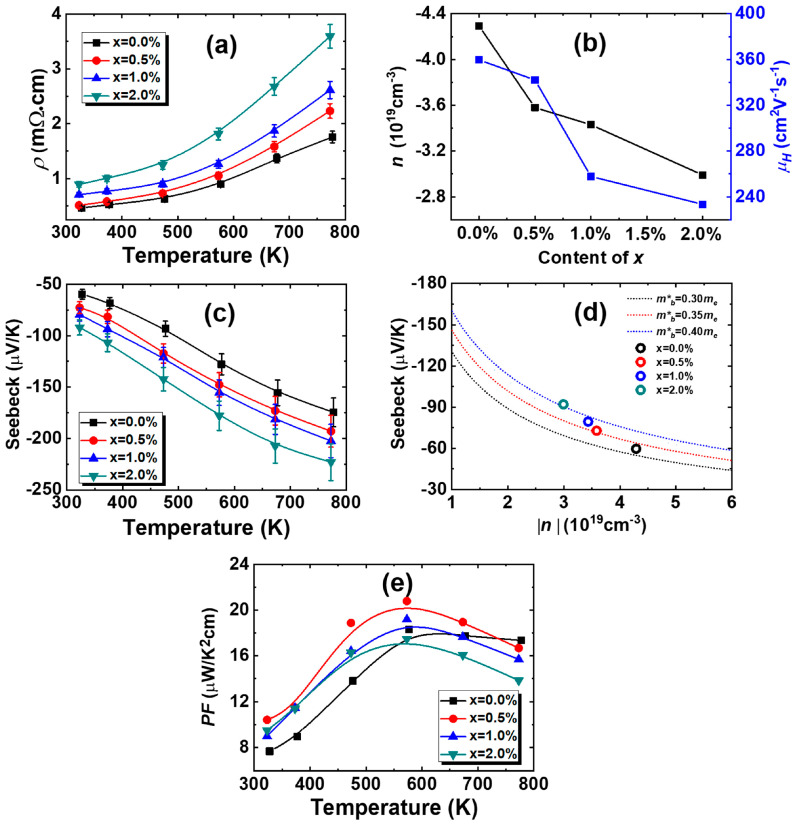
Temperature dependence of (**a**) electrical resistivity, (**c**) Seebeck coefficient, and (**e**) power factor for Pb_0.985−x_Sb_0.015_Mn_x_Te alloys. (**b**) The carrier concentrations as a function of doping concentration and (**d**) Seebeck coefficient as a function of carrier concentration for Pb_0.985−x_Sb_0.015_Mn_x_Te at room temperature.

Figure 3 presents the electrical characteristics of Pb_0.985−x_Sb_0.015_Mn_x_Te alloys. The electrical resistivity *ρ* rises as temperature rises, indicating that all alloys exhibit degenerate semiconductor behavior. With increasing Mn content, a general increase in electrical resistivity is observed. The *ρ* of the sample with x = 0.5% resistivity is 0.509 mΩ·cm at 323 K and rises to 0.893 mΩ·cm for the sample with x = 2.0%. To investigate the underlying reasons for this resistivity change, Figure 3b presents the carrier concentration *n* and mobility *μ_H_* at ambient temperature. Notably, all samples exhibit negative *n*, suggesting Pb_0.985−x_Sb_0.015_Mn_x_Te alloys are all n-type. With the rise of Mn, the *n* tends to decrease. Additionally, the *μ_H_* diminishes as the Mn augments, partially owing to the strengthened defect scattering resulting from the substitution of Mn atoms for Pb atoms. Another contributing factor is the inverse relationship between *μ_H_* and $m_{b}^{*}$, and Mn doping results in an increase in $m_{b}^{*}$ (which will be discussed later), leading to a decrease in *μ_H_*. In conclusion, the increasing trend of *ρ* with the increase in Mn content can be attributed to the combined influence of reduced *n* and *μ_H_* (*ρ* = 1/*nμ_H_*).

Figure 3c depicts the Seebeck coefficient *S*, revealing negative values across the entire temperature range for all samples. This indicates n-type conductive behavior, which aligns with the negative carrier concentrations. The absolute values of Seebeck coefficient |*S*| exhibit a nearly linear increase with temperature, which is characteristic of metallic behavior. Additionally, as the Mn doping increases, |*S*| rises. Specifically, when the Mn increases from 0.5% to 2.0%, the |*S*| at 323 K climbs from 72.8 to 92.1 μVK^−1^. The increase in the |*S*| is attributable not solely to the rise in *n* but also related to the $m_{d}^{*}$ of the energy bands, as evidenced by the relationship between *n* and *S* shown in Figure 3d. The dashed line is the theoretical Pisarenko line (SPB model) based on the following equations [33]:

(1) $$\begin{array}{l} n=\frac{4\pi {\left(2m_{d}^{*}k_{B}T\right)}^{3/2}}{h^{3}}F_{1/2}(\eta )\\ S=\frac{k_{B}}{e}\left[\frac{r+2}{r+1}\frac{F_{r+1}(\eta )}{F_{r}(\eta )}-\eta \right], \end{array}$$

$F_{n}(\eta )$ is Fermi integrals calculated by the following formula:

(2) $$F_{n}(\eta )=\int_{0}^{\infty }\frac{x^{n}}{1+\exp(x-\eta )}dx,$$

*η* is reduced Fermi level obtained through *η = E_F_*/*k_B_T*; $m_{d}^{*}$, h, and $k_{B}$ are effective mass, Boltzmann constant, and Planck’s constant; and *r* denotes the scatter factor, which is assumed to be 0 based on the dominance of the acoustic scattering mechanism [34,35]. The Pisarenko plots, depicted by black, red, and blue lines, have been theoretically computed with effective masses of 0.30, 0.35, and 0.40 *m_e_*. As the doping content of Mn increases, effective mass also increases, primarily due to the flattening of energy bands caused by Mn doping, which has been confirmed in other studies [24,26]. The power factor (*PF*), calculated by *PF* = *S*^2^/*ρ*, is plotted against temperature in Figure 3e. Despite Mn doping causing the increase in *ρ*, the *PF* of the samples is generally enhanced across the full temperature range, resulting from the substantial increase in the |*S*|. For x = 0.5%, the maximum power factor reaches about 20.8 μW/K^2^cm at 573 K.

**Figure 4:**
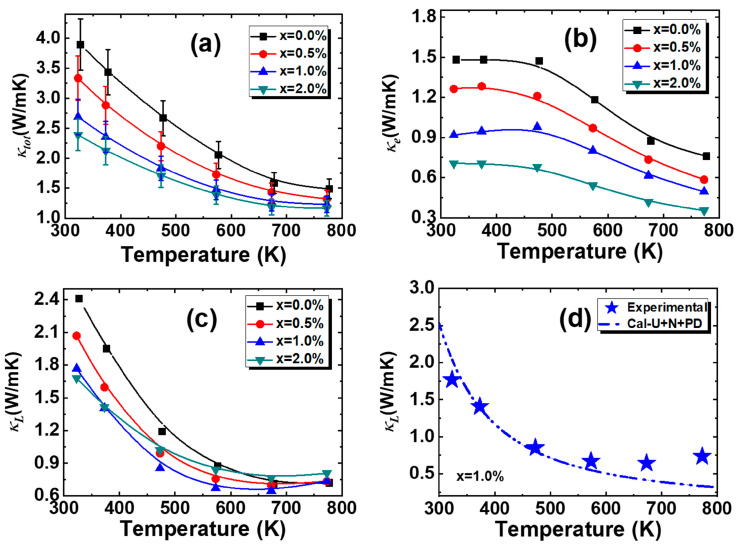
Temperature dependence of (**a**) the total thermal conductivity, (**b**) the electronic thermal conductivity, and (**c**) the lattice thermal conductivity for Pb_0.985−x_Sb_0.015_Mn_x_Te alloys. (**d**) The theoretical lattice thermal conductivity for the 1.0% Mn-doped alloy.

The thermal properties are illustrated in Figure 4. The *κ_tot_* decreases with the increasing temperature for all alloys, and with the increase in the content of Mn, *κ_tot_* exhibits a decreasing trend. Specifically, *κ_tot_* decreases from 3.89 W m^−1^ K^−1^ for the x = 0.0% sample to 2.38 W m^−1^ K^−1^ for the x = 2.0% sample at 323K. The *κ_e_* is estimated by Wiedemann–Franz law [36]. As a result of the elevated *ρ*, the *κ_e_* experiences a significant reduction. The *κ_L_* is obtained by the equation *κ_L_ = κ_tot_ − κ_e_*. As the temperature increases, the *κ_L_* of all alloys decreases, but it exhibits a minor rise at higher temperatures. With the increase in Mn doping content, the *κ_L_* of the alloys at room temperature decreases. Additionally, at medium to high temperatures, except for the sample with x = 2.0%, the *κ_L_* of the other samples also decreases. The lowest *κ_L_* of 0.64 Wm^−1^K^−1^ has been obtained for the 1.0% Mn-doped alloy at 673 K. To further investigate thermal conductivity, a fitting analysis based on the Debye–Callaway model is performed, and specific details can be found in our previously published reports [6]. For the 1.0% Mn-doped alloy, the experimental values of *κ_L_* align well with the theoretical values in the low-to-mid temperature range, which takes into account the influence of point defect introduced by Mn substituting for Pb. Therefore, the reduction in *κ_L_* is mainly attributed to the strengthened point defect scattering.

**Figure 5:**
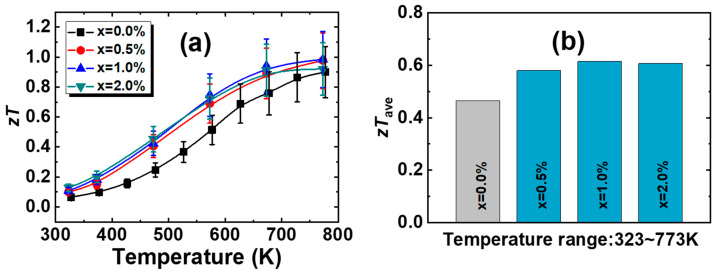
Temperature dependence of the (**a**) *zT* values and (**b**) *zT_ave_* values for Pb_0.985−x_Sb_0.015_Mn_x_Te alloys.

The figures of merit *zTs* for Pb_0.985−x_Sb_0.015_Mn_x_Te alloys are shown in Figure 5a. The peak values of the figure of merit (*zT_max_*) for nearly all the samples are achieved at 773 K. The *zTs* of the alloys have improved across the entire temperature range after Mn doping. The samples with x = 1.0% and 2.0% have achieved the highest *zT* value of 1.0, which is attributed to the improvement in the *PF* and the decrease in *κ_tot_*. Figure 5b shows the average figure of merit (*zT_ave_*) for Pb_0.985−x_Sb_0.015_Mn_x_Te alloys. The *zT_ave_* value of the sample with x = 0.0% is 0.46 (323–773 K). After the introduction of Mn, the *zT_ave_* values of the samples improved, ranging between 0.58 and 0.62. The 1.0% Mn_-_doped alloy exhibits the highest *zT_ave_*_,_ ~0.62, attributed to it possessing the highest *zT* across nearly the entire temperature range. The TGA curves depicted in Figure S1 show the weight change of the x = 1.0% sample as it is heated to 873 K in N_2_ at a rate of 10 K/min. The mass loss of the sample is only 0.5% at 873 K, which fully shows that the sample has excellent thermal stability. The above discussion indicates that Mn doping can synergistically optimize thermal and electrical performance, and this strategy holds great potential for enhancing the performance of other thermoelectric systems. However, despite the improvement in the thermoelectric performance of the n-type PbTe samples in this work, it remains relatively low compared to other studies [34,37,38,39]. This is primarily attributed to the suboptimal carrier concentration and limited phonon scattering mechanisms in the samples of this study. Further enhancements can be pursued by adjusting doping elements, compositing components, and optimizing preparation processes to optimize carrier concentration and introduce hierarchical phonon scattering mechanisms.

## 4. Conclusions

High-density Mn-doped n-type PbTe alloys with uniform element distribution have been synthesized by traditional solid-state reactions followed by hot-pressing. Mn doping synergistically optimizes the electronic and thermal transport properties of the alloys. For the former, the introduction of Mn enhances the effective mass and reduces the carrier concentration, leading to a significant increase in the absolute value of the Seebeck coefficient. Consequently, the power factor of the alloys is enhanced, with the maximum power factor reaching 20.8 μW/K^2^cm for the 0.5% Mn-doped sample at 573 K. For the latter, the strengthened point defect scattering contributes to a substantial reduction in lattice thermal conductivity, with the lowest lattice thermal conductivity achieving 0.64 Wm^−1^K^−1^ for 1.0% Mn-doped sample at 673 K. As a result, a notably enhanced *zT*_max_ of 1.0 at 773 K and *zT*_ave_ of 0.62 (323–773 K) are achieved in the 1.0% Mn-doped sample. These findings confirm that Mn doping can synergistically optimize the thermal and electrical properties of n-type PbTe, yet its zT value still awaits further enhancement through strategies such as optimizing carrier concentration and introducing hierarchical phonon scattering.

## Data Availability

The original contributions presented in the study are included in the article/Supplementary Material, further inquiries can be directed to the corresponding authors.

## Supplementary Materials

The following supporting information can be downloaded at https://www.mdpi.com/article/10.3390/ma18051029/s1, Figure S1: The TGA curves of Pb_0.985−x_Sb_0.015_Mn_x_Te (x = 1.0%) alloy heating to 873 K in N_2_ at a rate of 10 K/min.
