# Supplementary material for: Enhancing the Thermoelectric Performance of n-Type PbTe via Mn Doping
Source: Materials (Basel). 2025 Feb 26;18(5):1029. doi: 10.3390/ma18051029 (PMC11901316; doi:10.3390/ma18051029)
Supplement: Supplementary file 1 [file materials-18-01029-s001.zip › materials-3426095-supplementary.pdf]

Supplementary Materials

Enhancing the thermoelectric performance of n-type PbTe via Mn doping

Tingting Chen [1], Yaqi Shao [1], Ruilin Feng [1], Junxiang Zhang [1], Qidong Wang [1], Yanan Dong [1], Hongan Ma [2],
Bing Sun [1,*] and Dongwei Ao [3,*]

[1] School of Physics and Electronic Information, Weifang University, Weifang 261061, China; chentingting@wfu.edu.cn (T.C.); 15563423102@163.com (Y.S.); z969600069@163.com (R.F.); 15553612871@163.com (J.Z.); wangqidong2001@163.com (Q.W.); yndong@wfu.edu.cn (Y.D.)
[2] National Key Lab of Superhard Materials, Jilin University, Changchun 130012, China; maha@jlu.edu.cn
[3] School of Machinery and Automation, Weifang University, Weifang 261061, China
* Correspondence: wywdsunbing@wfu.edu.cn (B.S.); aodongwei@wfu.edu.cn (D.A.)

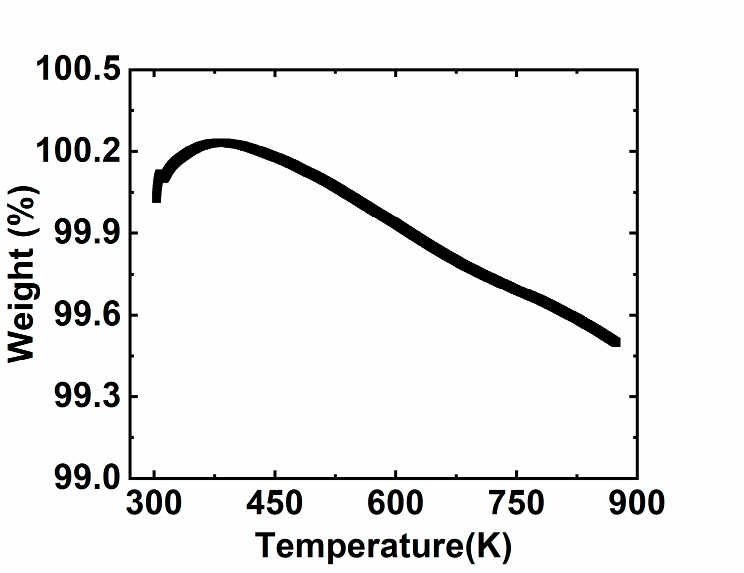

Figure S1 The TGA curves of $Pb_{0.985-x}Sb_{0.015}Mn_xTe$ (x=1.0%) alloy heating to 873 K in N_2 at a rate of 10 K/min
